# Gender and Age Differences in Lifestyle Factors Related to Hypertension in Middle-Aged Civil Service Employees

**DOI:** 10.2188/jea.13.38

**Published:** 2007-11-30

**Authors:** Yoko Hori, Hideaki Toyoshima, Takaaki Kondo, Koji Tamakoshi, Hiroshi Yatsuya, ShanKuan Zhu, Takashi Kawamura, Junji Toyama, Noboru Okamoto

**Affiliations:** 1 Department of Public Health/Health Information Dynamics, Nagoya University, Graduate School of Medicine.; 2 Current address: Obesity Research Center, Columbia University College of Physicians & Surgeons, New York, USA.; 3 Kyoto University Center for Student Health.; 4 Aichi Prefectural Owari Hospital.; 5 Aichi San-no-maru Hospital.

**Keywords:** cross-sectional study, hypertension, gender, preference for salty taste, body mass index

## Abstract

The aim of this study is to identify lifestyle factors related to hypertension in man and woman workers, and to investigate age and gender differences in the relationships of the factors. From 6,000 civil service employees (4,937 men and 1,063 women) aged 40-69 years, information on lifestyle-related factors such as stress, exercise habits, preference for salty taste, alcohol drinking and smoking habits, and body mass index, as well as age and family history of hypertension was obtained through self-administered questionnaires in 1997. Hypertension was defined as either a systolic blood pressure ≧ 140mmHg, a diastolic blood pressure ≧ 90 mmHg, or undergoing treatment for hypertension, and was present by 37.0% in men and 19.6% in women. Only body mass index was a significant lifestyle-related risk factor common to both genders with an odds ratio and its 95% confidence interval in parentheses of 2.2 (2.0 - 2.5) for men and 3.2 (2.3 - 4.6) for women. Men and women who preferred salty taste showed multivariate adjusted odds ratios of 0.9 (0.8 -1.1) and 1.5 (1.1 - 2.2) for hypertension, respectively. In the stratified subanalysis, women aged 50 years and over had a significant odds ratio of 2.7 (1.5 - 4.9), whereas women aged 40-49 years and men of all age classes failed to show significant relationships. Salt intake was suggested to be a key factor for hypertension particularly for women after menopause.

Among lifestyle-related diseases, hypertension is a major risk factor for cardiovascular diseases in both men and women.^1^ Because hypertension has a high detection rate in annual health check-ups, it has become an important illness from the standpoint of health care of employees. Epidemiologic studies performed in Western countries as well as in Japan have clearly shown that there was a gender difference in the morbidity of hypertension.^2^^,^^3^ Some studies performed in Western countries have also shown further that the morbidity of hypertension in women increases after menopause.^4^^,^^5^ This finding attracts attention from the viewpoint of health guidance for hypertension in middle-aged women. In occupational health field in Japan, however, the health strategies for women have been set to protect the motherhood, and they did not pay enough attention to life-style related diseases.^6^ Therefore, we started collection of annual health check-up data of middle-aged civil service employees and made a questionnaire survey on their lifestyle in 1997.

In the present study, we did a cross-sectional analysis of the baseline data of annual health check-ups and a questionnaire survey on lifestyle to determine the difference between men and women in the lifestyle factors related to hypertension, and also to investigate differences due to age and gender in their relationships.

## METHODS

### Study population

The subjects were 8,560 local government employees (7,046 men and 1,514 women), aged 40 years and more as of April 1, 1997. The survey was done using a self-administered questionnaire with a total of 44 items related to lifestyle, including exercise, diet, and stress. The questionnaires were distributed to the subjects in advance of their annual health check-up, and were collected on the day of the check-up. The health check-ups were conducted during the period from April 1997 through February 1998. Answers to the questionnaire and written consent to view health check-up data were obtained from 5,062 men and 1,075 women (response rate: men 71.8%, women 71.0%). Sixty-seven of them (61 men, 6 women) had a history of myocardial infarction or stroke, or were taking medications for these diseases, and were excluded from the analysis due to the possibility that their lifestyles had already changed. Seventy who were currently normotensive without taking antihypertensives (64 men, 6 women) were also excluded on the grounds that they had a history of hypertension.

In the analysis, the hypertension group (1,825 men and 208 women) was defined as including (1) those with a systolic blood pressure ≧ 140 mmHg, or a diastolic pressure ≧ 90 mmHg, or (2) those undergoing treatment for hypertension regardless of blood pressure levels. For comparison, a healthy control group (3,112 men and 855 women) was formed of those with a systolic blood pressure < 140 mmHg and diastolic pressure < 90 mmHg without a history of hypertension. A total of 6,000 people (4,937 men and 1,063 women) were ultimately subjected to analysis.

### Blood pressure measurements

Blood pressure was measured by auscultation using a mercury sphygmomanometer in 66.0% of the subjects analyzed, and by an automated sphygmomanometer (UM-15, Parama-Tech Co., Japan) in 34.0%. For both measurement methods, Korotkoff phases 1 and 5 were taken as the systolic and diastolic blood pressure, respectively. In the automated equipment, monitoring by oscillometric method was used in combination to deal with artificial noises. Both systolic and diastolic blood pressures measured by this method have been shown to agree with those obtained by invasive method.^7^

As a rule, measurements were taken from the right arm with the subjects in a sitting position after a minimum of 5 minutes of rest. If the first measurement values were outside the normal range, the measurements were repeated after the subject sat quietly for several more minutes. Since only the lower values among the measurements were recorded in the health check up card, they were used in this study.

### Questionnaire

Six lifestyle factors out of nine listed in the chapter ‘Lifestyle Modifications for Hypertension Prevention and Management’ of the Sixth Report of the Joint National Committee on Prevention, Detection, Evaluation and Treatment of High Blood Pressure (JNC VI^8^) were used in this analysis among the items in life style questionnaire. They were stress, exercise habits, preference for salty taste, alcohol drinking habits, smoking habits, and body mass index (BMI). Preference for salty taste was substituted for salt intake.

Subjects were classified into two groups according to their response to the question, ‘How much daily stress do you feel?’ on the questionnaire. Subjects who answered ‘very much’ or ‘fairly much’ were combined to form the ‘high stress’ group, while those who answered ‘the usual’ or ‘little’ were combined into the ‘low stress’ group. Subjects were also grouped according to their answer to the question, ‘Do you normally exercise or engage in sports other than work?’ Those who responded that they exercised one or more times a week were placed in an ‘exercise habit’ group, and those who exercised one to three times per month or almost never in a ‘no exercise habit’ group.

As to a preference for saltiness, subjects were classified into three groups based on their response to the question, ‘Do you like food strongly seasoned with salt, soy sauce, and *miso* (soybean paste)?’ Response choices were ‘prefer salty taste and often eat foods so seasoned’, ‘prefer salty taste but abstain from foods so seasoned’, ‘prefer bland taste’, or ‘no special preference’. Those who chose either one of the first two answers were combined into a ‘prefer salty taste’ group, and this group was compared with the other two groups.

Those responding that they consumed alcohol once or more a week were classified into an ‘alcohol habit’ group, while those who consumed alcohol ‘almost never’ or ‘not at all’ were listed as a ‘no alcohol habit’ group. Those who responded that they smoked were placed in a ‘smoking habit’ group, and those who had quit smoking or never smoked were combined into a ‘no smoking habit’ group.

BMI was calculated from the height and weight measurements in the health check-up using the formula ‘weight (kg)/square of height (m)’, and was used as the indicator of obesity. Subjects were classified into two groups according to BMI with a cutoff value of 24.0 (kg/m^2^), the integer closest to the former standard^9^ for being overweight of the Japan Society for the Study of Obesity.

In addition to these lifestyle habits, a family history of hypertension and age were included as factors for analysis. The family history was judged based on information on history of hypertension in both parents and siblings. Subjects were grouped by age into four classes of 40-44, 45-49, 50-54, and 55 years and older.

### Statistical analysis

The relationship between hypertension and each factor was investigated using a chi-square test. The Cochran-Armitage method was used to test the trend of proportion. In a comparison among the three groups, the significance level was corrected using Bonferroni’s methods. To investigate items having independent relations with hypertension even after adjusting for confounding among factors, a multivariate logistic regression analysis was employed separately for men and women, with lifestyle factors, age class, and family history of hypertension as independent variables. For the preference for saltiness and age classes, dummy variables were assigned by taking ‘prefer bland taste’ and ‘40-44’ as the respective references. The strength of the relationship between the dependent variable and each of the independent variables was expressed by the odds ratio (OR) and its 95% confidence interval (CI). The statistical analysis package SPSS^®^ 10.0J for Windows was used for statistical processing. The significance level was set at p<0.05.

## RESULTS

Table 1 shows the distribution of subjects with hypertension by gender and age. The proportion of hypertensives was significantly lower in women than in men overall (19.6% vs 37.0%, p<0.05), as well as for every age class. The proportion increased significantly (p<0.001) with the advance of age in both men and women.

**Table 1.** Distribution of the hypertensives by gender and age class.

	age (years)					trend test^†^

	40-44	45-49	50-54	55-	all ages	
			Men			
Number of subjects	1,140	1,809	966	1,022	4,937	
						
Number of hypertensives	336	614	398	477	1,825	
(%)	29.5*	33.9*	41.2*	46.7*	37.0*	p<0.001
Number of subjects under treatment of hypertension	35	120	107	172	434	
(%)	3.1	6.6	11.1	16.8	8.8	p<0.001

			Women			
Number of subjects	332	393	170	168	1,063	
						
Number of hypertensives	44	65	44	55	208	
(%)	13.3	16.5	25.9	32.7	19.6	p<0.001
Number of subjects under treatment of hypertension	8	17	16	25	66	
(%)	2.4	4.3	9.4	14.9	6.2	p<0.001

* : p<0.05 vs women by *χ*^2^ test

† : Cochran-Armitage test

Table 2 shows the distribution of each factor for hypertensive and healthy men, as well as the multivariate OR of hypertensives and its 95% CI. In addition to the age class, factors showing a significant positive association with an OR ≧ 1.0 were, in order of decreasing OR, ‘BMI ≧ 24.0’, ‘positive family history of hypertension’, and ‘drinking habit’. Factors showing a significant negative association were ‘no special preference for salt’ and ‘smoking habit’.

**Table 2.** Relationship between hypertension and investigated factors for it in men.

Factor	Number (%) of		*χ*^2^ value	Odds ratio ^†^(95% CI)

	Hypertensives	Controls		
	(1,825 in total)	(3,112 in total)		
Age (years)			83.3***	
40-44	336 (18.4)	804 (25.8)		1.0
45-49	614 (33.6)	1,195 (38.4)		1.2 (1.0-1.4)
50-54	398 (21.8)	568 (18.3)		1.6 (1.4-2.0)
55-	477 (26.1)	545 (17.5)		2.0 (1.7-2.5)
				trend p<0.001
Body mass index (kg/m^2^)			175.5***	
<24.0	935 (51.3)	2,183 (70.1)		1.0
≧ 24.0	889 (48.7)	929 (29.9)		2.2 (2.0-2.5)

Preference for salty taste			18.4***	
Prefer bland taste	490 (27.0)	803 (25.9)		1.0
No special preference	228 (12.5)	531 (17.1)		0.7 (0.6-0.9)
Prefer salty taste	1,100 (60.5)	1,769 (57.0)		0.9 (0.8-1.1)

Stress			0.004	
Low	965 (53.2)	1,649 (53.1)		1.0
High	849 (46.8)	1,459 (46.9)		1.0 (0.9-1.2)

Exercise			0.3	
No	1,282 (70.8)	2,176 (70.1)		1.0
Yes	529 (29.2)	930 (29.9)		0.9 (0.8-1.1)

Alcohol Drinking habit			16.0***	
No	418 (23.2)	876 (28.4)		1.0
Yes	1,387 (76.8)	2,204 (71.6)		1.3 (1.2-1.5)

Smoking habit			9.3***	
No	1,040 (59.8)	1,649 (55.3)		1.0
Yes	698 (40.2)	1,335 (44.7)		0.8 (0.7-0.9)

Family history of hypertension			120.8***	
No	1,057 (57.9)	2,277 (73.2)		1.0
Yes	767 (42.1)	835 (26.8)		2.0 (1.8-2.3)

***: p<0.001

CI: confidence interval

† : Confounding effects between the factor in question and all other factors on the odds ratio were adjusted simultaneously through a multivariate logistic regression analysis.

Subjects with unknown response were excluded.

Table 3 shows the results for women. In addition to age class, the factors showing a significant positive association with hypertension were ‘BMI ≧ 24.0’, ‘positive family history’, and ‘prefer salty taste’. No factor showed a significant negative association.

**Table 3.** Relationship between hypertension and investigated factors for women.

Factor	Number (%) of		*χ*^2^ value	Odds ratio ^†^(95% CI)

	Hypertensives	Controls		
	(208 in total)	(855 in total)		
Age (years)			33.5***	
40-44	44 (21.2)	288 (33.7)		1.0
45-49	65 (31.3)	328 (38.4)		1.0 (0.6-1.5)
50-54	44 (21.2)	126 (14.7)		1.7 (1.02-2.8)
55-	55 (26.4)	113 (13.2)		2.4 (1.5-3.9)
				trend p<0.001
Body mass index (kg/m^2^)			58.7***	
<24.0	123 (59.1)	715 (83.6)		1.0
≧ 24.0	85 (40.9)	140 (16.4)		3.2 (2.3-4.6)

Preference for salty taste			15.5***	
Prefer bland taste	79 (38.0)	416 (48.9)		1.0
No special preference	34 (16.3)	169 (19.9)		1.0 (0.7-1.7)
Prefer salty taste	95 (45.7)	266 (31.3)		1.5 (1.1-2.2)

Stress			0.3	
Low	79 (38.0)	344 (40.3)		1.0
High	129 (62.0)	510 (59.7)		1.0 (0.7-1.5)

Exercise			0.05	
No	154 (75.1)	647 (75.8)		1.0
Yes	51 (24.9)	206 (24.2)		1.0 (0.7-1.5)

Alcohol Drinking habit			1.7	
No	139 (68.1)	531 (63.0)		1.0
Yes	65 (31.9)	312 (37.0)		0.9 (0.6-1.3)

Smoking habit			0.4	
No	189 (96.4)	788 (95.1)		1.0
Yes	7 (3.6)	41 (4.9)		0.8 (0.4-1.9)

Family history of hypertension			11.9**	
No	108 (51.9)	557 (65.1)		1.0
Yes	100 (48.1)	298 (34.9)		1.8 (1.3-2.6)

**: p<0.01 ***: p<0.001

CI: confidence interval

† : Confounding effects between the factor in question and all other factors on the odds ratio were adjusted simultaneously through a multivariate logistic regression analysis.

Subjects with unknown response were excluded.

The OR of age class, treated with a dummy variable, was significant in all age classes for men, whereas it was significant only in the two age classes above 50 years for women (Tables 2 and 3).

**Figure 1.**
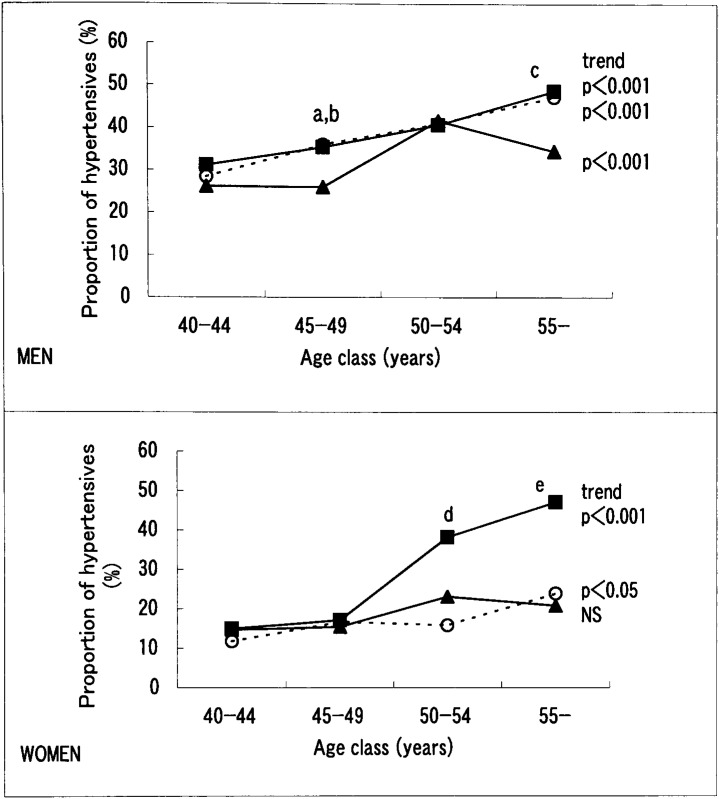
Proportion of hypertensives according to age class, gender, and preference for salty taste. —■— prefer salty taste; ···○···prefer bland taste; —▲— no special preference The result of the Cochran-Armitage trend test showed a significant increase in the proportional rate of hypertensives with advancing age in men preferring a salty taste (p<0.001), men preferring a bland taste (p<0.001), women preferring a salty taste (p<0.05), and women preferring a bland taste (p<0.001). In women, the proportion of hypertensives among those who preferred a salty taste increased remarkably after the age of 50, and was significantly higher than that of hypertensives among those who preferred a bland taste. a: p<0.05, prefer salty taste vs. no special preference, b: p<0.01, prefer salty taste vs. no special preference, c: p<0.05, prefer salty taste vs. no special preference, d: p<0.05, prefer salty taste vs. prefer bland taste, e: p<0.01, prefer salty taste vs. prefer bland taste

To investigate the causes that brought about such differences between men and women in the relationships of the proportion of hypertensives with a preference for salty taste and age class, we compared the proportion of hypertensives among the three groups which differed in the preference for salty taste in each age class (Figure 1). Among men, the proportion of hypertensives showed a statistically significant increase with age in all three groups (p<0.001 for the ‘prefer salty taste’ and ‘prefer bland taste’ groups, and p<0.01 for the ‘no special preference’ group). However, there was no significant difference in this proportion between the group which preferred salty taste and the group which preferred bland taste in any age class, although the proportion was significantly lower in the group with ‘no special preference’ than in the other two groups for the age classes 45-49 (p<0.05 for prefer bland taste group and p<0.01 for prefer salty taste group) and 55 and over (p<0.05).

Among women as well, the proportion of hypertensives in three groups increased with age, though the increase was significant only in the ‘prefer salty taste’ group (p<0.001) and ‘prefer bland taste’ group (p<0.05). It is noteworthy that the increase in the proportion of the ‘prefer salty taste’ group was remarkable for those over 50 years of age (Figure 1). A comparison of the proportion of hypertensives among the three taste groups in each age class revealed no significant difference for women in their 40s, whereas in the two age classes above 50, the proportion of hypertensives in the ‘prefer salty taste’ group was significantly higher than in the ‘prefer bland taste’ group (p<0.01 for 50-54 years and p<0.05 for 55 years and over, respectively). The ‘no special preference’ group showed an age-related trend of proportion similar to that of the ‘prefer bland taste’ group.

To confirm the above differences according to gender and age by adjusting for the confounding effects of other factors, a multivariate logistic regression analysis was done for groups of men and women in the age classes of the 40s and 50 and over (Table 4). The adjustment factors were the same as those used in Tables 2 and 3 except for age. Age was also included as a continuous variable in each model to adjust for the age effect within each age class. As a result, ‘prefer salty taste’ showed a significant positive relation with hypertension only among women aged 50 years and above, with the relation being similar to that shown in Figure 1.

**Table 4.** Relationship between hypertension and the preference for salty taste by gender and age (odds ratios and their 95% confidence intervals).

Preference for salty taste	Men		Women	
	
	40-49 years	50 years and older	40-49 years	50 years and older
			
	(n=2,823)	(n=1,861)	(n=707)	(n=315)
Prefer bland taste	1.0	1.0	1.0	1.0

No special preferenc	0.7 (0.5-0.9)	0.8 (0.6-1.1)	1.0 (0.6-1.8)	1.3(0.5-3.0)

Prefer salty taste	0.9 (0.8-1.1)	0.9 (0.8-1.2)	1.0 (0.6-1.6)	2.6(1.4-4.6)

trend	p=0.64	p=0.75	p=0.93	P=0.001

n: number

Numbers in each cell are odds ratio and 95 % confidence interval (in parentheses) adjusted for body mass index, stress, exercise habit, alcohol drinking habit, smoking habit, family history of hypertension and age.

## DISCUSSION

The aim of this study was to clarify the differences between men and women workers in factors related to hypertension. Among lifestyle related factors, the one that had a positive significant relation in both genders was BMI ≧ 24.0. In epidemiologic surveys conducted in the 1950s through the 1970s in Japan, the development of hypertension was found to be mainly related to a high salt intake,^10^ but unrelated to obesity.^11^ However, with the increased consumption of animal food products and other improvements in lifestyle,^12^ obesity has also become a risk factor related to hypertension.^13^ In our study as well, among the lifestyle factors investigated, obesity was one and only factor significantly related to hypertension in both men and women.

The positive relationship in this study of hypertension to family history of hypertension, common to men and women, provides additional information that the strength of this relationship is almost the same in both men and women. To the best of our knowledge, this was not shown in past reports,^14^^,^^15^ since both genders were analyzed as a group.

Specific to men were a positive association of drinking habits and a negative association of smoking habits with hypertension. A linear relationship between the levels of alcohol consumption and blood pressure has been reported from studies with Japanese men,^16^^,^^17^ and a significant positive correlation was seen in the present study as well. For Japanese women, in contrast, there have been no reports showing such a relationship, which differs from findings among women in Western countries.^18^^,^^19^ The present study also failed to identify such a relationship, most likely because of the scarcity of women alcohol drinkers (36%) compared with American women (54%)^18^ and the small amount of alcohol consumed.

As once explained in the guidelines proposed in JNC IV^20^ and V,^21^ no causality between smoking and essential hypertension has been determined except for acute effect. However, there are even several cross-sectional studies showing a negative correlation between the two.^22^^,^^23^ In the present study, the proportion of male non-smokers in the control group was 21.0%, lower than 25.0% in the hypertension group. It is most likely that intensive advice to quit smoking for hypertensives by health care takers would have resulted in such rate difference.^22^ It has been indicated that the rate of female smokers in Japan, less than 5% in the present study, is low compared with the global level.^24^ Therefore, our sample size would have been too small to confirm a biological relationship between smoking and hypertension in women.

Preference for salty taste showed different associations with hypertension according to gender and age. The associations between salty taste preference and hypertension became stronger in the women of the age groups 50 years and older, but it was not apparent in the women groups younger than that or in men. According to the Intersalt Study,^25^ the greater the urinary sodium excretion, the steeper was the slope of blood pressure with age. We are not certain to what degree the preference for salty taste represented salt intake in our population, since we did not measure salt intake or salt excretion in the urine. However, our findings in older women seem to agree with those in the Intersalt Study, as long as the preference reflects the salt intake. The lack of such associations in men would be related to findings by others^26^^-^^28^ on sensitivity for salt. They reported that the preference and intake of salt were consistent in women but not in men or that ability to distinguish the taste of saltiness was higher in women than in men.^29^ Thus, it is possible that the different consistency of taste and intake between the two genders have caused a part of gender-difference of the associations.

Considering facts that the mean age of menopause of Japanese women has been reported about 50.5 years^30^ and the associations with salty taste preference were prominent in the age groups after 50 years, we have to think over the possible role of menopause in this association. Although no report has been found on the relationship between menopause and hypertension as to Japanese women to the best of our knowledge,^6^^,^^31^^,^^32^ both significant^33^^,^^34^ and insignificant^35^ epidemiologic results have been reported in the Western countries. Further, there are experimental studies^36^^,^^37^ showing a remarkable rise in blood pressure in ovariectomized Dahl salt-sensitive rats as well as clinical studies^38^ suggesting that decreases in sex hormones and increased sensitivity to sodium are related the genesis of postmenopausal hypertension. Given these reports, it is possible that the age-related associations in this study might have been related to menopause. However, it remains to be confirmed by further studies.

Lifestyle factors showing no relationship with hypertension in either gender were ‘much stress’ and ‘exercise habit.’ These findings do not dispute the effects of these factors on blood pressure levels so far proposed. To understand the true relationships, it will be necessary to conduct analyses that take account of different cut-off points or information for these factors, i.e., coping for stress,^39^ intensity of exercise,^40^ and/or stage of hypertension. Furthermore, it should have been necessary to measure blood pressure without distinction between normotensives and hypertensives. However, for the latter, the measurement was done twice and lower value was adopted. It is probable that this measurement bias would have yielded weaker relationship between lifestyle factors and hypertension than real one.

The present study has some limitations. We could not obtain information on actual salt intake or salt excretion in the urine, or information on menopause from individual subjects of this study. This renders our interpretation on the relationship among salt intake, menopause, and blood pressure speculative, although it is consistent with past experimental, epidemiologic and clinical findings. Furthermore, since this analysis was cross-sectional and the results do not necessarily explain the cause and effect relationships between the various lifestyle factors and hypertension. However, presence of gender and age-specific factors related to hypertension suggests a necessity for further study to look for efficient preventive approach.
